# Neonatal mortality and its associated factors among neonates admitted at public hospitals, pastoral region, Ethiopia: A health facility based study

**DOI:** 10.1371/journal.pone.0242481

**Published:** 2021-03-17

**Authors:** Abay Woday Tadesse, Yohannes Mekuria Negussie, Setognal Birara Aychiluhm

**Affiliations:** Department of Public Health, College of Medical and Health Sciences, Samara University, Afar Region, Samara, Ethiopia; Federal University of Sergipe, BRAZIL

## Abstract

**Background:**

Neonatal mortality is a public health issue in, Ethiopia. Unfortunately, the issue is noticeably under-reported and underestimated, so the true gravity of the situation cannot be acknowledged in developing regions of the country. Regrettably, there is no single study to show the rates and predictors of neonatal mortality for Afar region. Thus, this study aims to assess neonatal mortality and associated factors in Afar region, Ethiopia.

**Methods:**

A health facility-based cross-sectional study was conducted on 403 neonates admitted to the neonatal intensive care units (NICUs) from January 2015 to December 2019. Maternal and neonatal medical records were reviewed and audited using structured data extraction checklist. The data was collected by four trained nurses and midwives. The medical records were selected using a systematic random sampling technique. Bivariate and multivariable logistic regression analyses were done. Adjusted odds ratio with the corresponding 95% confidence interval were used to assess the association between neonatal mortality and the associated factors. Finally, the statistical significance level was declared at a p-value of less than 0.05.

**Results:**

In this study, 391 medical records of newborns were included with the data complete rate of 97.02%. The prevalence of neonatal mortality was 57 (14.6%) [95% CI 11.0%-18.4%]. A multivariable logistic regression showed that lack of antenatal care (ANC) follow up [AOR = 4.69: 95%CI (1.77, 12.47)], giving birth through cesarean section [AOR 3.59, 95%CI (1.22, 10.55)], having admission temperature less than 36.5°C [AOR 10.75, 95%CI (3.75, 30.80)], birth asphyxia [AOR 7.16, 95%CI (2.22, 23.10)], and having a length of stay greater than five days in the hospital [AOR 0.23, 95%CI (0.08, 0.66)] were significantly associated with neonatal mortality.

**Conclusion:**

This study revealed that the rate of neonatal mortality is still high compared to the national data. Antenatal care, cesarean section delivery, length of stay in the hospital, low temperature at admission and birth asphyxia were factors associated with neonatal mortality. Thus, the health facilities should give due attention to improve antenatal care, intrapartum care and standardized care for admitted neonates. Furthermore, prospective studies are recommended.

## Introduction

The World Health Organization (WHO) defines neonates as live-born infants whose age is within 28 complete days of birth [1]. Neonatal mortality (NM) is defined as infant death, which occurred during the first four weeks of life after birth [1–3].

Analysis of Demographic and Health Surveys from 25 sub-Saharan African countries from 2000 to 2016 showed that the percentage of all live births occurring in health facilities ranged from 22% to 92% [4]. In Ethiopia, below fifty percent (48%) of pregnant women gave birth at health facilities in 2019 [5].

Between 1990 and 2017, the global Neonatal Mortality Rate (NMR) decreased by 51%, from 36·6 deaths per 1000 livebirths in 1990, to 18.1 deaths per 1000 livebirths in 2017 [6]. In 2018, an estimated 2.5 million neonatal deaths happened worldwide. Of these, more than 1.1 million deaths were contributed by sub-Saharan African countries [7,8]. In Ethiopia, neonatal mortality rate ranges between 23.4 deaths per 1000 live births and 44 deaths per 1000 live births [9–12]. Though Ethiopia has made great progress in the reduction of neonatal mortality rates from 2000 (49 per 1000 live births) to 2019 (30 per 1000 live births), the neonatal mortality reduction in Afar region is at stable state (45 per 1000 in 2000 and 39 per 1000 live births in 2019) compared to other developed regions of the country [5,12,13].

Studies conducted in developing countries have been identified various risk factors associated with neonatal mortality. These include maternal education level, multiple births, lack of antenatal cares, maternal infections during pregnancy, prematurity, birth asphyxia, neonatal sepsis [14–18]. Moreover, studies conducted across Ethiopia have been identified different causes of neonatal deaths. Of these, 67% of neonatal deaths were attributed to birth asphyxia, neonatal infections and prematurity respectively [19–23].

Globally, different strategies, and policies have been tried to reduce neonatal, infant and under five mortalities. These include Millennium Development Goals (MDG-4) [24] that was implemented to reduce child mortality by three-fourth at the end of 2015. The second is the Sustainable Development Goal (SDGs-3) which has 13 specific targets to reduce the burden of neonatal mortality [i.e. 20 to 12 deaths per 1000 live births] by the end of 2030 [25,26]. In Ethiopia, the Federal Ministry of Health developed a national strategy for addressing maternal and newborn health with the primary health care approach and health extension package implemented since 1990s [27,28]. Besides, newborn’s health was deemed one of Ethiopia’s top priorities in the past ten years as an indicator of country’s development index. The country has also designed the newly revised child survival and newborn care strategy (2016–2020) [3,24,29–31]. Beyond these efforts, Afar region, one of the emerging regions in Ethiopia, has still the highest neonatal mortality rate (38 deaths per 1000 live births) [12] compared to developed regions of the country. Moreover, local studies conducted in developed regions of the country revealed important subnational variations in mortality rates and causes, which might be masked by estimation methods at the national level. Therefore, this study was intended to assess the prevalence and associated factors of neonatal mortality in Afar region.

## Methods and materials

### Study settings and participants

A health facility-based cross-sectional study was conducted to assess the prevalence of neonatal mortality and its associated factors among neonates admitted in NICUs (i.e. Dubti referral and Aysaita district hospitals). This study was conducted to retrieve a five years data, from January 2015 to December 2019. Afar region has the total population of 1.6 million. Both of the study hospitals had neonatology units since 2005. The NICUs are equipped with functional incubators, oxygen devices, 5–8 admission beds, 4–6 trained nurses and at least one pediatrician for each. The NICUs of the hospitals gave service for more than 2000 cases during the five years. All of the neonates admitted in the health facilities of the region were considered as source population while all neonates admitted in the selected hospitals were considered as the study population.

All live born neonates who were born in the selected hospitals, less or equal to 28 days, admitted into the two neonatal intensive care units (NICUs) of public or government hospitals for any reasons from January 2015 to December 2019 were eligible for this study. However, all neonates whose discharge summary sheet did not clearly show whether they were alive or died, and neonates immediately referred to specialized health facilities for further management were excluded from this study. Because these neonates were difficult to measure their outcomes. Besides, the medical records of the mothers were also traced to address maternal factors (i.e. Parity, gravidity, ANC follow up for the current birth, residence, age, and medical problems during pregnancy) that was reviewed from delivery log-books.

The sample size was determined for both objectives (i.e. to determine the prevalence of neonatal mortality and to identify factors associated with neonatal mortality). Then, the maximum sample size which was determined for the first objective was used for this study. Therefore, the required sample size was calculated using a single population proportion formula. Since we do not have studies conducted in developing regions of the country to represent the proportion of neonatal mortality in this regions, we have taken 50% of the proportion of neonatal mortality (P = 50%) with the basic assumptions of 95% confidence interval (the Critical value Zα/2 = 1.96), 5% margin of error and the researchers added 5% to compensate the incomplete data. Then, the calculated sample size became 403 (384**×**0.05 + 384).

$$n=\frac{{\left(Za/2\right)}^{2}(P)(1-P)}{d^{2}}=384$$

Where: **n** = the required sample size, **Z α/2** = the standardized normal distribution curve value for the 95% confidence interval, **P** = the proportion of neonatal mortality among the general newborns, and **d** = degree of precision (the margin of error between the sample and population) (**See Table 1**).

**Table 1:** Sample size determination for the 2nd objective (determinants of neonatal mortality) and 1st objective, 2019.

Predictor Variables	Assumptions: power = 80%, r = 2, & 95% confidence	Studies	Sample size	5% NRR	Final Sample size
**Prematurity**	P unexposed = 20%	Kolobo, et al. 2017, [32]	N1 = 37	**6**	**Nf = 117**
	P exposed = 48%		N2 = 74		
No ANC follow-ups for this birth	P unexposed = 26.3%	Kidus et al. 2018, [33]	N1 = 31	5	Nf = 98
	P exposed = 58.7%		N2 = 62		
**Proportion of NNM**	**P = 50**	NA	N0 = 384	19	**Nf = 403**

There were five hospitals in Afar region. Of these, two hospitals were selected purposely since these are the only hospitals which have functional intensive care units to care neonates after admission. The sample size was proportionally allocated into the selected hospitals based on their total neonatal admission rates of the previous one-year of the study period. The two hospitals had 2,015 total neonatal cases (N) admitted in the last five years (i.e. 1105 neonates admitted in Dubti hospital and 910 neonates admitted in Ayisaita hospital) that gave sample of 182 and 221 neonates, respectively. To calculate the required number of participants from each hospitals, we multiplied the total number of neonates admitted in each facility by the sampling fraction (n/N). The sampling fraction was equal to five for the two hospitals. Then, index medical record of newborn was selected using a lottery method from 1 to 5 in each hospital, which was number three. Accordingly, every 5^th^ participants were selected using systematic random sampling technique from the admission registration book in the order of 3^rd^, 8^th^, 13^th^, 18^th^, … etc until the required sample size was reached. Besides, the corresponding maternal records were also retrieved from the delivery log-books.

### Data collection tools and procedures

The data extraction checklist was adapted from studies conducted in Ethiopia [10,23,34] and it was modified after checking the NICU admission log-book and delivery registration books. The chart review checklist was developed in English language. It consists sociodemographic characteristics, maternal factor**s (**age, ANC follow up, mode of delivery, place of delivery, maternal HIV status, parity, multiple delivery, gravidity, residence and medical illness during pregnancy), fetal factors (sex, birth weight, status at birth, diagnosis of disease, gestational age, APGAR scores at birth, neonate’s HIV status) and health care providers related factors (medication given at admission, vital signs taken at admission, schedule for the provision of the prescribed medications).

Training was provided over two days for data collectors and supervisors. The checklist was pretested on 5% of the samples on records before January 01, 2015. Then, a panel of experts verified content validity of the instruments, and the required revisions were made.

Data was collected by trained midwifes and nurses who were working in non-selected hospitals and health centers (Samara and Logia health centers) using a standardized, pretested and structured reviewer administered checklist. The data collectors had first reviewed medical record of the neonate based on the inclusion criteria. Then, they had also traced the medical records of the corresponding mothers as soon as they finish the review of the neonates.

### Definitions

Completed data: a data that has at least complete discharge summary about the admitted neonate including possible cause of death.

Neonate: a new born baby within 28 days of life.

Antenatal Care (ANC): if the mothers have visited health facility at least once for antenatal care for this index birth (current newborn), we have considered them as they have ANC visits (Yes/no) irrespective of the number of visits.

Birth asphyxia: defined as failure to initiate and sustain normal breathing at the first and fifth minute of birth.

### Data processing and analysis

The collected data were coded, checked for completeness, and cleaned for errors. Then, data were entered into Epi-Data version 3.1 and exported into SPSS version 23.0 for statistical analysis. The assumptions for normality were checked. The descriptive statistics was done and the results were presented using texts, tables, mean and standard deviation. The bivariable logistic regression analysis was done to identify variables those have a p-value of less than 0.25 to be considered in the multivariable logistic regression analysis model. Correlation between independent variables was checked using variance inflation factors (VIF). The model fitness was assessed using Hosmer-Lemeshow goodness test. Thus, we have used clinical significance of predictor variables, absence of multi-collinearity between independent variables, variables with p-value <0.25 in the bivariate analysis and model adequacy to select and enter independent variables into the final model.

Then, multivariable logistic regression analysis was done to identify factors associated with neonatal mortality. The findings of the final model were reported using adjusted odds ratios with the corresponding 95% Confidence Interval. Finally, a statistically significant level was declared at a p-value <0.05.

### Ethical considerations and consent for participants

Ethical clearance was obtained from ethical review board (ERB) of Samara University, College of Medical and Health Sciences with Ref. No. CMHS/38/35/124/19. Then, a permission letter was written from the Regional Health Bureau to the purposely-selected Hospitals. Finally, an official permission letter was obtained from each hospital to proceed with the data collection. Informed consent was not applicable for the study since the data was collected with medical chart review. Confidentiality was maintained by keeping records in a secured manner and avoiding personal identifiers.

## Results

### Study participants sociodemographic characteristics

A total of 403 neonatal medical charts were reviewed with 97.02% data complete rate. In this study, 251 (64.2%) of the admitted neonates were males and 317 (81.1%) of the neonates were admitted into the neonatal intensive care unit (NICU) at less than 7 days of age. The mean age of mothers was 26.05 (SD±5.35) and 321 (82.1%) of mothers resided in urban areas (**see Table 2**).

**Table 2:** Sociodemographic characteristics of study participants admitted in NICU, Afar region, 2019.

Characteristics	Response	Frequency	Percentage
**Sex of neonate**	Male	251	64.2
	Female	140	35.8
**Age of neonate at admission (in days)**	≤7	317	81.1
	≥7	74	18.9
**Residence of mother**	Urban	321	82.1
	Rural	70	17.9
**Age of the mother**	15–19	36	9.2
	20–24	142	36.3
	25–29	113	28.9
	30–34	67	17.1
	35+	33	8.4
**Number of cases admitted and year of admission at NICU**	2015	39	10.0
	2016	86	22.0
	2017	83	21.2
	2018	104	26.6
	2019	79	20.2

### Maternal health related conditions

The maternal chart review revealed that more than two thirds of the mothers [239 (68.7%)] had four or more ANC visits during their pregnancy. On the other hand, only 43 (11%) of mothers had no ANC follow up at all. The majority of (328 (83.9%)) the mothers gave birth through spontaneously vaginal delivery. Of the total mothers, 284 (72.6%) of them had a history of multigravida, only 27 (6.9%) of them had multiple delivery, and only 13 (3.3%) of the mothers had known recorded medical illnesses during pregnancy (**see Table 3**).

**Table 3:** Maternal health related factors in selected hospitals from May 2015 to May 2019, Afar region.

Characteristics	Response	Frequency	Percentage
**Gravidity**	Primi gravida	107	27.4
	Multi gravida	284	72.6
**Parity**	Primi para	68	17.4
	Multi para	323	82.6
**ANC follow up**	Yes	348	89.0
	No	43	11.0
**Mode of delivery**	SVD	328	83.9
	Assisted vaginal delivery	33	8.4
	C/S	30	7.7
**Multiple delivery**	Yes	27	6.9
	No	364	93.1
**Chronic medical illness**	Yes	13	3.3
	No	378	96.7
**HIV status of the neonate at birth**	Non-exposed	381	97.4
	Exposed	10	2.6

### Fetal health related conditions

The neonatal chart review found that 93 (23.5%) of neonates were preterm and 118 (30.2%) of them had a birth weight less than 2.5 kilo grams. In addition, 270 (69.1%) of the neonates had a resuscitation history at birth and only 10 (2.6%) of the admitted neonates were from HIV positive mothers. The leading causes of admission were; early onset neonatal sepsis (43.5%), low birth weight (27.1%) and prematurity (23.5%) (**See Table 4**).

**Table 4:** Fetal health conditions and causes of neonatal admission in NICU from May 2015 to May 2019 (n = 391), Afar region, Ethiopia.

List of variables	Category of variable	Frequency	Percentage
**Gestational age (in weeks)**	Preterm (<37)	93	23.5
	Term (≥37)	299	76.5
**Weight at birth (in kg)**	< 2.5	118	30.2
	≥2.5	274	70.1
**Temperature at admission (in degree cellcious)**	36.5–37.5	137	35.0
	< 36.5	46	11.8
	> 37.5	208	53.2
**Resuscitation given at birth**	Yes	270	69.1
	No	121	30.9
**HIV/AIDS status of neonate**	Negative	381	97.4
	Positive	10	2.6
**Causes of neonatal admission in the NICU**	Prematurity	93	23.5
	Very low birth weight	10	2.3
	Low birth weight	108	27.6
	Early onset neonatal sepsis	171	43.7
	Late onset neonatal sepsis	56	14.3
	Birth asphyxia	24	6.1
	Respiratory distress syndrome (RDS)	58	14.8
	Hypoglycemia	5	1.3
	Neonatal jaundice	12	3.1
	Severe birth trauma	13	3.3
	RVI exposed	10	2.3
	Congenital malformation	9	2.3
	Others*	5	1.3

Others* = Hepatitis infection^1^, anemia^2^ and necrotizing enterocolitis^2^. ***Note*:** One neonate may have more than one causes of admission.

### Neonatal admission cares

**Fig 1:**
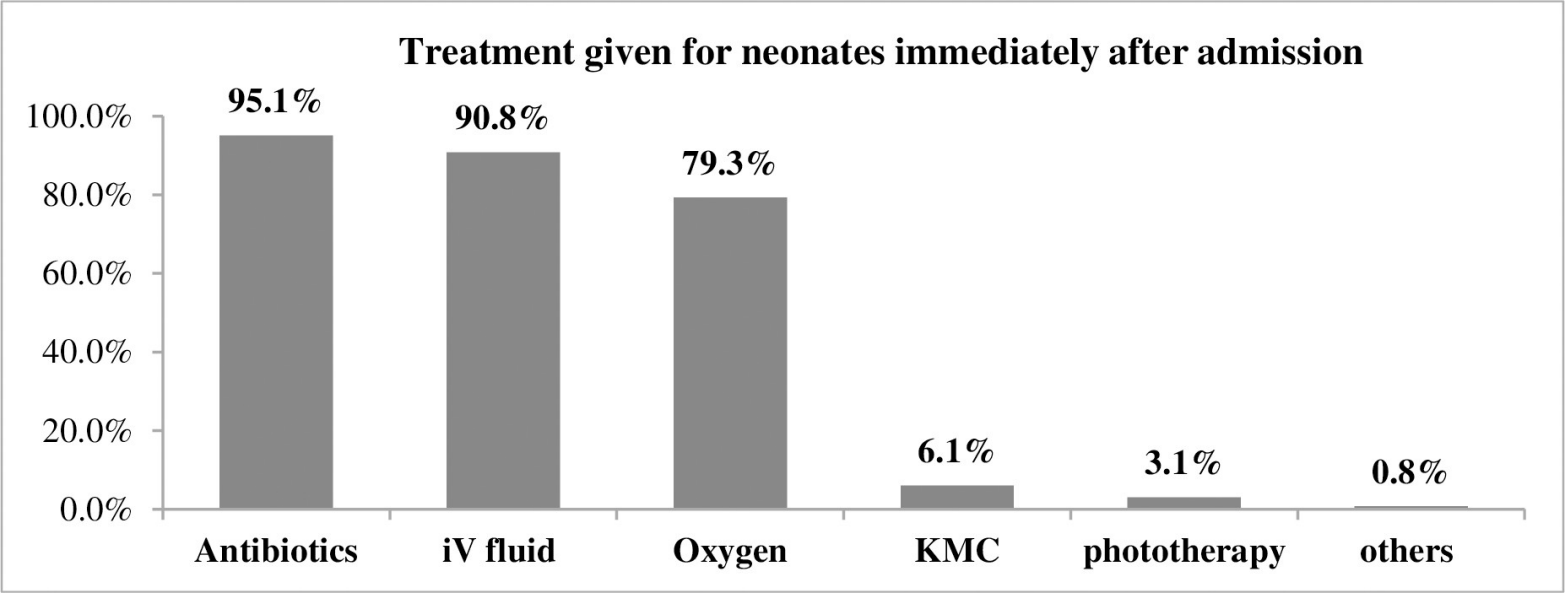
Cares given for neonates admitted in NICU from May 2015 to May 2019, Afar, Ethiopia.

In this study, 372 (95.1%) and 355 (90.8%) of the admitted neonates were given antibiotics and IV fluids containing glucose within one hour of admission respectively. In addition, 310 (79.3%) of neonates were supplemented with oxygen and 12 (3.1%) of them were given phototherapy. Here, we reassured that one neonate may be given more than one care immediately after admission (**see Fig 1**).

### Neonatal outcomes at discharge and causes of neonatal mortality

**Fig 2:**
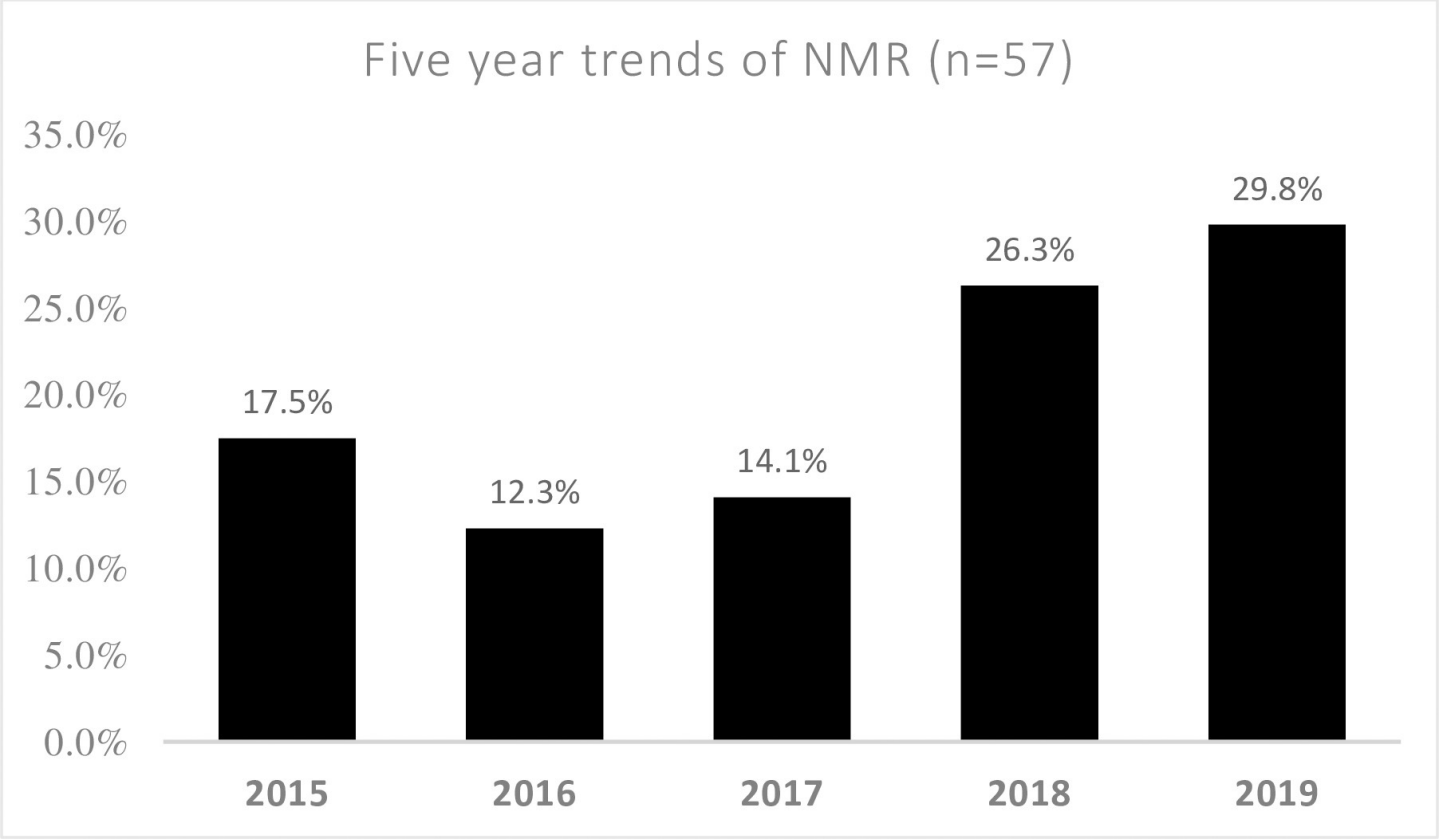
Trends of neonatal mortality from 2015 to 2019 among neonates admitted in NICU, Afar, Ethiopia.

In this study, 57 (14.6%) [95% CI 11.0%-18.4%] of neonates admitted in the NICUs were died within 28 days of life. The study revealed that 300 (76.7%) of the neonates were discharged within 5 days of admission and the mean length of stay in NICUs was 4.16 days ±3.07 SD. In this study, the leading causes of neonatal mortality were prematurity (43.9%), early onset neonatal sepsis (35.1%), low birth weight (33.4%) and birth asphyxia (21.1%) (**See Table 5**). When we look the five years trend of neonatal mortality, it is still steadily increasing from 2015 [10(17.5%)] to 2019 [17(29.8%)] (**See Fig 2**).

**Table 5:** Outcomes of admission and causes of neonatal mortality among neonates admitted in NICU from May 2015 to May 2019, Afar region, Ethiopia.

List of variables	Category of variable	Frequency	Percentage
**Discharge outcome**	Alive	334	85.4
	Death	57	14.6
**Total stay in NICU (in days)**	< 5 days	300	76.7
	≥ 5 days	91	23.3
**Causes of neonatal mortality (n = 57)**	Prematurity	25	43.9
	Low birth weight	19	33.4
	Early onset neonatal sepsis	20	35.1
	Late onset neonatal sepsis	8	14.0
	Perinatal asphyxia	12	21.1
	Respiratory distress syndrome (RDS)	13	22.8
	Hypothermia	6	10.5
	Neonatal jaundice	6	10.5
	Severe birth trauma	3	5.3
	RVI exposed	3	5.3
	Others*	6	10.6

Others* = NEC^2^, Congenital anomalies^1^, anemia^1^, hypoglycemia^1^ and hyperthermia^1^. **Note:** One neonate can have more than one cause.

### Factors associated with neonatal mortality

After checking the model for multi-collinearity and model fitness; variables with a p-value < 0.25 in the bivariate analysis and clinically important variables were considered in the final model. Hence, the multivariable logistic regression analysis showed that lack of ANC follow up, giving birth through cesarean section, having admission temperature less than 36.5°C, being asphyxiated at birth and length of stay in the NICU for less than five days after admission were the independent predictors of neonatal mortality.

Neonates born from mothers who did not receive ANC follow up during pregnancy had 4.7 times a greater odds of neonatal mortality compared to neonates born from mothers who had ANC follow up [AOR = 4.69: 95%CI (1.77, 12.47)]. The odds of neonatal mortalities among neonates born through cesarean section was 3.6 times greater compared to neonates born through spontaneous vaginal delivery [AOR = 3.59: 95%CI (1.22, 10.55)]. Neonates who were admitted because of birth asphyxia had 7.1 times a greater odds of neonatal death compared to those who were not asphyxiated at all [AOR = 7.16: 95%CI (2.22, 23.10)]. The odds of neonatal death among neonates who had a body temperature less than 36.5 at admission was 10.7 times higher compared to neonates who had a normal body temperature at admission [AOR = 10.75: 95%CI (3.75, 30.80)]. Furthermore, neonates stayed for five or more days at NICU were 77% less likely to die compared to neonates who stayed for less than five days [AOR = 0.23: 95%CI (0.08, 0.66)] (**see Table 6**).

**Table 6:** Factors associated with neonatal mortality among neonates admitted in NICU, Afar region, 2019.

List of variables	Category of variables	Neonatal death		COR 95% CI	AOR 95% CI	P-values for AOR
		Yes	No			
**Sex of neonate**	Male	42(16.7)	209 (83.3)	1.68(0.89,3.14)	1.63(0.74,3.58)	0.362
	Female	15(10.7)	125(89.3)	1.00	1.00	
**ANC follow up**	Yes	41 (11.8)	307 (88.2)	1.00	1.00	0.000
	No	16 (37.2)	27 (62.8)	4.43 (2.21, 8.93)	4.69 (1.77,12.47)	
**Mode of delivery**	SVD	40(12.2)	288(87.8)	1.00	1.00	
	Assisted delivery	7(21.2)	26(78.8)	1.94 (0.79,4.76)	3.00 (0.10,9.06)	0.124
	C/S	10(33.3)	20(66.7)	3.6 (1.57,8.24)	3.59 (1.22,10.55)	0.022
**Multiple birth**	Yes	9(33.3)	18(66.7)	3.29 (1.40,7.75)	2.15 (0.71,6.50)	0.223
	No	48(13.2)	316(86.8)	1.00	1.00	
**Gestational age at birth (in weeks)**	Preterm (<37wks)	25(26.9)	68(73.1%)	3.06 (1.70,5.50)	1.77 (0.78,4.04)	0.225
	Term & above	32(10.7)	266(89.3%)	1.00	1.00	
**Temperature at admission**	36.5–37.5	10 (7.3)	127(92.7%)	1.00	1.00	
	< 36.5	23(50.0)	20(50.0)	12.7 (5.35,30.17)	10.75 (3.75,30.80)	0.000
	> 37.5	24(11.5)	184 (88.5)	1.66 (0.77,3.58)	1.57 (0.64,3.82)	0.275
					
**Death due to birth asphyxia**	Yes	11(45.8%)	13(54.2%)	5.9 (2.50,13.96)	7.16 (2.22,23.10)	0.000
	No	46(12.5%)	321(57.5%)	1.00	1.00	
**Death due to RDS**	Yes	5 (38.5%)	8(61.5%)	3.92 (1.23,12.44)	1.60 (0.32,8.03)	0.308
	No	52(13.8%)	326(86.2%)	1.00	1.00	
**Length of stay**	< 5 days	50(16.7%)	250(83.3%)	1.00	100	0.008
	≥ 5 days	7 (7.7%)	84(92.3%)	0.417 (0.18,0.95)	0.231 (0.08,0.66)	

**Key:** COR = Crude Odds Ratio, AOR = Adjusted Odds Ratio, C/S = Cesarean Section, SVD = Spontaneous Vaginal Delivery, RDS = Respiratory Distress Syndrome.

## Discussions

In developing countries like Ethiopia, rates of neonatal mortality are several folds higher compared to developed nations, which ranges 23.4 to 44 per 1000 live births [9–12]. Furthermore, the magnitude of neonatal mortality is optimally high in Afar regions compared to the overall national mortality rate (38 and 29 per 1000 live births respectively) [12]. Beyond the national survey, accurate epidemiological data is scarce and the exact magnitude of neonatal mortality in pastoral communities of Ethiopia is not clearly known. This study revealed that the overall prevalence of neonatal mortality in the Afar region is still optimally high. The multivariable logistic regression found that lack of ANC follow up, neonates delivered by caesarean section, neonates who had a body temperature of less than 36.5 ^o^C at admission, neonates who stayed less than five days in the NICU and neonates with birth asphyxia were the independent predictors of neonatal mortality in the Afar region.

In this study, the prevalence of neonatal mortality was14.6% which is similar to studies conducted in Gondar university teaching hospital, Northwest Ethiopia (14.3%) [23] and Felege Hiwot referral hospital, Bahir Dar (13.3%) [34]. However, the result of this study is higher than the prevalence of neonatal mortality reported in the Somali region, Ethiopia (5.7%) [35], a study done in Jimma zone, Southwest Ethiopia (3.2%) [21], a study done in North Gondar, Northwest Ethiopia (4.4%) [10], and a study done in Ayder referral hospital, Mekelle (6.6%) [20]. On the other hand, the prevalence of neonatal mortality in this study is lower than a study conducted in Mizan Tepi university teaching hospital (22.8%) [9], and a study conducted in Gondar university teaching hospital (23.1%) [19]. The differences could be explained by the existence of sociocultural and socio-economic differences across Ethiopian regions regarding health service utilization, differences in hospital set-ups (equipment available and skilled persons). Besides, there will be differences in awareness of the community upon utilization of available health services like delivery at health facilities, and visiting health facilities for sick neonates and children.

In this study, prematurity (43.9%), early onset neonatal sepsis (35.1%), perinatal asphyxia and low birth weight (21.1%) were the major causes of neonatal mortality in the region. This finding is similar to a study conducted in Mizan Tepi university teaching hospital that revealed prematurity (31%), neonatal sepsis (29.7%), low birth weight (15.3%) and birth asphyxia (7.7%) as the leading causes of death [9]. This could be explained by the majority of neonatal deaths in developing countries are related to conditions of labor, intrapartum and the immediate newborn care practices. From this, it can be seen that neonatal survival interventions are not targeting the intrapartum as well as immediate and early neonatal periods, and as a direct result, neonatal mortality has not declined in the needed manner.

In this study, the odds of neonatal mortality was 5-folds higher among mothers who did not have ANC follow-up compared to those mothers did have ANC follow-ups. This finding is similar with a study conducted in North Shoa zone, Amhara region [36], and a study conducted in Jimma zone, Southwest Ethiopia [21]. This can be explained by the fact that women not having antenatal care follow up during pregnancy are more at risk for pregnancy and intrapartum related problems, which in turn, can put the newborn at risk of death. Therefore, women with adequate antenatal care visits have a better chance of early detection and management of the birth related problems.

This study revealed that neonates stayed for five or more days at NICU were 77% less likely to die compared to neonates who stayed for less than five days. This finding is in line with a study conducted in Jimma university medical center which stated that neonates who stayed for less than seven days in the NICU had 3.9 times higher risk of neonatal mortality when compared to those who stayed for seven or more days [37]. This can be explained because most neonatal deaths happen in the early neonatal periods (0–6 days of life) than in the late neonatal period of life (7–28 days) [22,23]. Consequently, it was found that neonatal mortality was related to the hospital length of stay. Thus, due attention should be given for neonates in the early neonatal period to reduce neonatal mortality in the health facilities.

In this study, the odds of death among neonates who had a body temperature less than 36.5 ^0^C at admission was 10.7 times higher compared to those neonates who had a normal body temperature at admission (36.5–37.5^o^c). This is consistent with a retrospective cohort study conducted in southern Ethiopia referral hospitals which state that neonates who had a temperature of less than 36.5 at admission had higher risk of death than neonates with normal temperature (36.5 to 37.5) [38]. This can be justified by neonates who are in a hypothermic state may be more prone to different infections. As result, they are more likely to become septic and die when compared to neonates with normal body temperatures.

This study indicated that neonates delivered by cesarean section had 3.6 times higher odds of neonatal death than neonates born spontaneously by vaginal (SVD). This finding is similar with a study conducted at Pakistan, where delivery using C-section had increased risk of neonatal mortality [39]. This might be related to neonates born via C-section without clear indications such as prolonged labor, fetal distress, obstructed labor and other medical problems during pregnancy. These neonates delivered through C-section are at greater risk of birth asphyxia than neonates born with birth canal. Therefore, neonates born through C-section had a high probability of death than neonates delivered through the natural birth canal. However, this result is contrary with a study conducted in southern Ethiopia referral hospital NICU, where neonates delivered using C-section had 66% less chance of risk of death compared to SVD [38]. This can explained by timely decision making rather than simply waiting for vaginal delivery, which may save the life of the neonate and the mother. Thus, delivering through C-section with clear indications can reduce the risk of death by early identification and intervention of birth related complications such as prolonged labor.

This study revealed that neonates who were admitted because of birth asphyxia had 7.1 times a greater odds of neonatal death compared to those who were not asphyxiated at all This is consistent with a study conducted in southern Ethiopia referral hospitals [38], which found that neonates with birth asphyxia had 2 times higher risk of death than their counterparts. This finding is also similar with a study conducted in Jimma university medical center, which revealed neonates who had a history of birth asphyxia had 5 times greater risk of death [37]. This may be due to the fact that besides commencement of adequate efforts after admission, neonates with respiratory problems like birth asphyxia had a greater risk for a poor prognosis and death compared to neonates admitted with other medical problems. Therefore, neonates with a respiratory distress have higher chance of death when compared to those who do not experience any respiratory distress.

### Study limitations

The study was included the medical records of the neonates and their mothers that was found in the selected hospitals, which may not display all factors pertaining to neonatal mortality. So, the results may not be fully representative of the community’s neonatal mortality. In addition, there will be a potential to misclassification of neonatal deaths due to blame of health care providers for neonatal mortality after admission. Thus, this may under estimate the prevalence of neonatal mortality in health facilities.

## Conclusion

This study revealed that the rate of neonatal mortality is still high compared to the national data. Antenatal care, cesarean section delivery, length of stay in the hospital, low temperature with in the first hour of admission and perinatal asphyxia were factors associated with neonatal mortality. Thus, the health facilities should give due attention for improving antenatal cares, intrapartum cares and standardized cares for admitted neonates. A prospective studies are recommended.

## Supporting information

S1 Dataset SPSS dataset, 2019. (SAV)

## Abbreviations

ANC: Antenatal Care
AOR: Adjusted Odds Ratio
MDG: Millennium Developmental Goal
NICU: Neonatal Intensive Care Unit
NMR: Neonatal Mortality Rate
RDS: Respiratory Distress Syndrome
WHO: World Health Organization
